# Upregulated hsa_circ_0005785 Facilitates Cell Growth and Metastasis of Hepatocellular Carcinoma Through the miR-578/APRIL Axis

**DOI:** 10.3389/fonc.2020.01388

**Published:** 2020-08-19

**Authors:** Anqi Wu, Yi Li, Mingzhu Kong, Baihui Zhu, Ruoyu Liu, Fang Bao, Shaoqing Ju, Lin Chen, Feng Wang

**Affiliations:** ^1^Department of Laboratory Medicine, Affiliated Hospital of Nantong University, Nantong, China; ^2^Department of Laboratory Medicine, School of Public Health, Nantong University, Nantong, China; ^3^Department of Gastroenterology and Laboratory Medicine, Nantong Third Hospital Affiliated to Nantong University, Nantong, China

**Keywords:** hepatocellular carcinoma, circular RNA, hsa_circ_0005785, miR-578, APRIL

## Abstract

Although accumulating documents have expounded the pivotal position of circular RNAs (circRNAs) in hepatocarcinogenesis and progression, the overwhelming majority of their functions and molecular mechanisms in hepatocellular carcinoma (HCC) are elusive. Herein, we explored the functions and potential mechanisms of hsa_circ_0005785 in HCC, which was aberrantly overexpressed in HCC and related to HCC patients' TNM stage and overall survival. Moreover, hsa_circ_0005785 depletion could repress proliferation and metastasis of the HCC cell *in vitro*, lead to cell apoptosis and cell-cycle arrest, and restrain HCC cell growth *in vivo*. Furthermore, mechanism analyses discovered that hsa_circ_0005785 adsorbed miR-578 by playing a miRNA sponge role, which resulted in the derepression of a proliferation-inducing ligand (APRIL) expression, miR-578's mRNA target. Besides, hsa_circ_0005785 reversed the suppressive influence of miR-578 on HCC and accelerated tumor malignant progression through the miR-578/APRIL axis. Taken together, our current study revealed an oncogenic role of hsa_circ_0005785 in the tumorigenesis of HCC. Moreover, targeting to the hsa_circ_0005785/miR-578/APRIL regulatory pathway might be a promising diagnostic and therapeutic strategy for HCC clinical practice.

## Introduction

As one of the most frequent malignancies, hepatocellular carcinoma (HCC) has high morbidity, recurrence, and mortality rate all over the world, especially in China. Despite of the huge progress in hepatectomy, radiotherapy, and chemotherapy, HCC patients still have been suffering from a poor survival rate during the past few decades, owing to the lack of reliably sensitive early diagnostic biomarkers (1, 2). Therefore, discovery of new biomarkers is critically needed to ameliorate the clinical diagnosis and prognosis of HCC patients. Up to now, plenty of documents have exposed that most of the non-coding RNAs, for example miRNA and lncRNA, are always dysregulated in HCC and play important roles in hepatocarcinogenesis (3–5). Yet, as a special category of non-coding RNAs, circular RNAs (circRNAs) need to further clarify the expression profile, clinical performance, and molecular mechanisms in HCC.

CircRNAs are derived from mRNA precursors and characterized by the covalently closed-ring single strand without 5′ cap or 3′ poly A tail structure (6, 7). CircRNAs functionally interact with RNA regulatory proteins (8), adjust the stability and expression of mRNAs (9), and have the potential of translation (10). Moreover, their studies have shown that circRNAs mainly localized in the cytoplasm and conduct as miRNA sponges or competing endogenous RNAs (ceRNAs), thereby modulating the miRNA's target, which are related to various biological processes (11, 12). In addition, mounting literatures have verified that circRNAs are frequently dysregulated and participated in the oncogenesis and progression of the malignancies through diverse mechanisms, disclosing the complexity in tumor pathogenesis, including HCC (13–15).

Herein, a fresh circRNA, named hsa_circ_0005785, was found drastically upregulated in HCC. What is more, it could play an oncogenic role in accelerating HCC malignant progression. Mechanism analyses uncovered that hsa_circ_0005785 could upregulate a proliferation-inducing ligand (APRIL) level by competitively binding miR-578 and topple the miR-578's suppressive effects on the growth and metastasis of HCC. Collectively, this study highlights the role of the hsa_circ_0005785/miR-578/APRIL axis in HCC, indicating the potential value of the circRNA-oriented clinical diagnosis and therapy for this deadly disease.

## Methods and Materials

### Sample Collection

Sixty HCC patients suffered from hepatoma resection, and their tumor tissues and paired adjacent normal tissues were gathered during January 2015 to December 2016 from the Affiliated Hospital of Nantong University. No patients have accepted any treatment prior to the operation. All patients' clinical pathological parameters were listed, as shown in Supplementary Table 1. After hepatectomy, tissues were rapidly snap-frozen in liquid nitrogen for further analysis.

### Cell Culture

Four HCC cell lines (Huh7, Hep3B, SK-Hep1, and HCCLM3) and LO2 (a normal human liver cell line) were bought from the Cell Resources Center of the Chinese Academy of Science. Cells were maintained in DMEM, contained with 10% fetal bovine serum (Gibco, USA), cultured with 5% CO_2_ at 37°C.

### Microarray Analysis

Five HCC tumor tissues and their adjacent normal tissues were enrolled to screen dysregulated circRNAs by a circRNA gene chip. RNase R was applied to eliminate linear RNA (Epicenter, USA). After being transcribed into the fluorescent-labeled cRNA, Arraystar Human circRNA Array V2.0 (8 × 15K, Arraystar, USA) was used to hybridize cRNA. Agilent Scanner G2505C System was then used to scan the slides (Agilent, USA).

### qRT-PCR Analysis

The RNA extraction reagent was used to separate total RNA (Thermo Fisher Scientific, USA) and reversely transcribed with PrimeScript RT polymerase (Takara, China). The levels of circRNA, miRNAs, and mRNA were detected with a Plus SYBR real-time PCR mixture (BioTeke, Beijing, China) by using LightCycler^®^ 480 qRT-PCR instrument (Roche, Germany). The formula 2^−ΔΔCt^ was utilized to compute the relative levels of circRNA, miRNA, and mRNA expression. 18S rRNA was used as an internal control for circRNA and mRNA. Moreover, U6 was used as an internal control for miRNAs. The detailed sequences of all primers were provided as a list in Supplementary Table 2.

### Plasmid Construction and Cell Transfection

HCC cells were incubated at about 80% confluence in 6-, 12-, and 96-well plates; the pcDNA3.1 vector (Invitrogen, USA), shRNA vector (GeneChem, China), or pMIR luciferase report vector (Ambion, USA) was transfected in a serum-free medium by using Lipofectamine 3000 (Thermo Fisher Scientific, USA). Then, DMEM containing 10% FBS was applied to replace the old cell culture media after 6 h of incubation. The cell functional experiments were performed after another 24–72 h of incubation. The shRNA sequences targeting hsa_circ_0005785, APRIL, and non-targeting control were listed, shown in Supplementary Table 3.

### Luciferase Reporter Experiment

HCC cells were treated with pMIR-circ-WT, pMIR-circ-Mut, pMIR-APRIL-3′UTR-WT, or pMIR-APRIL-3′UTR-Mut, together with miR-578 mimic or miR-NC, respectively. The activities of luciferase were examined with GloMax 20/20 Luminometer (Promega, USA) after 48 h of transfection.

### RNA Immunoprecipitation (RIP) Experiment

HCC cells were treated with RIP analysis reagent following the kit instructions (Millipore, USA); the RNA was immunoprecipitated against the Ago2 antibody. Hsa_circ_0005785 and miR-578 expressions were analyzed with qRT-PCR, with normal mouse IgG as a negative control.

### Cell Viability Assay

HCC cell viability was tested with CCK-8 kit (Dojindo, Japan) at different hours after transfection in 96-well plates. The optical density values were measured with Elx800 Reader (Bio-Tek, USA) at 450 nm after 2 h of incubation.

### Flow Cytometry Analysis

HCC cells were treated with RNase A and propidium iodide (Invitrogen, USA) for cell-cycle distribution and treated with Annexin V-FITC and propidium iodide for cell apoptosis analysis. Flow cytometric experiments were performed by FACSCalibur (BD, USA).

### Wound Healing Analysis

Six-well plates were used to culture HCC cells for 24 h. The wound gaps were generated via a 10-μL plastic pipette tip, and cells were incubated for another 24 h. The wound width was monitored by an inverted microscope for assessing the capacity of HCC cell migration.

### Transwell Assay

HCC cells in DMEM without serum were seeded in the top chamber, which was laid with a matrigel-coated membrane (BD, USA). Then, DMEM containing 10% FBS was filled in the low chamber for incubation for another 24 h. Cells were fixed on the surface of a low chamber with methanol, dyed using crystal violet for assessing the capacity of HCC cell invasion.

### Tumorigenicity in the Xenograft Model

SK-Hep-1 cells (1 × 10^7^) were injected into the ventral side subcutaneous tissue of each 4-week-old BALB/c nude mouse. The tumor volumes were examined weekly until anatomy. Tumor bodies were gathered and stained by immunohistochemistry analysis.

### Western Blot Experiment

Proteins of HCC cells were isolated with RIPA protein extraction reagent (Beyotime, China). After SDS-PAGE and transmembrane electrophoresis were conducted, the membrane was incubated with the first antibody targeting APRIL and β-actin (Santa Cruz, CA) overnight at 4°C. Then, the secondary antibody was added for 2 h at room temperature. Signal detection was performed with chemiluminescence imaging system (BIO-RAD, USA).

### Immunohistochemistry

Paraformaldehyde solution was used to fix the tumors. Then, paraffin imbedding was performed and tumors were cut into 4-μm-thick tissue sections. After being deparaffinized, antigen retrieval was performed for the slides by microwave treatment. The slides were incubated with the PCNA antibody and the secondary antibody (Santa Cruz, CA). After being washed, DAB staining and counterstaining with hematoxylin were performed.

### Statistical Analysis

In this study, all statistical analyses were carried out with SPSS 20.0 software. *P* < 0.05 was regarded as statistical significance. Differences of hsa_circ_0005785, miR-578, and APRIL levels in tissues were determined by non-parametric Mann–Whitney *U*-test. The association between hsa_circ_0005785 level and clinicopathological parameters was analyzed by Fisher's exact test. Student's *t*-test and one-way analysis of variance (ANOVA) were used for comparison among groups in the study of functions and mechanisms. Overall survival of HCC patients was carried out by the Kaplan–Meier method.

## Results

### hsa_circ_0005785 Was Overexpressed in HCC

**Figure 1:**
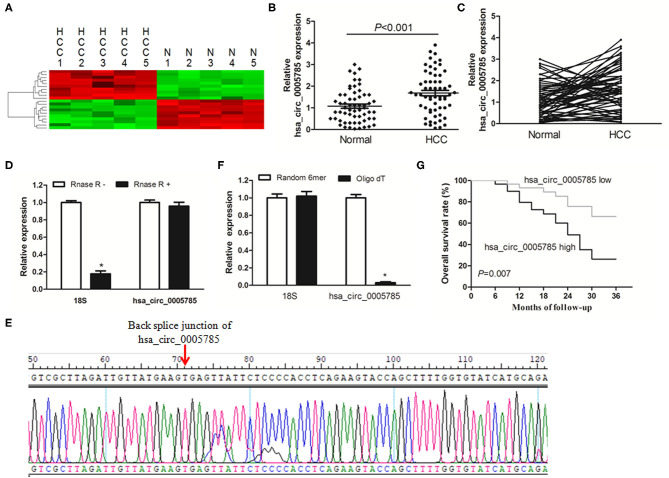
The relative expression of hsa_circ_0005785 in HCC tissues. **(A)** Heat map analysis of the top 10 obviously dysregulated circRNAs which resulted from microarray assays (5 HCC tissues vs. 5 paired normal tissues). **(B,C)** qRT-PCR detection of hsa_circ_0005785 expression in 60 HCC tissues and paired normal tissues. **(D)** Total RNA of HCC tissue was pretreated or not pretreated with RNase R; hsa_circ_0005785 and 18S rRNA levels were detected by qRT-PCR. **(E)** The qRT-PCR product of hsa_circ_0005785 was verified by Sanger sequencing. **(F)** The cDNA of hsa_circ_0005785 and 18S rRNA was reverse transcribed with random 6-mer or oligo dT primers and then measured by qRT-PCR. **(G)** The Kaplan–Meier curve analyzed the prognosis of HCC patients.

CircRNA microarray was conducted to screen the dysregulated circRNAs from 5 paired HCC tissues and corresponding normal tissues. In Figure 1A, the top 10 significantly dysregulated circRNAs were shown by using unsupervised hierarchical clustering analysis. Among them, we further verified that hsa_circ_0005785 expression was obviously raised in 60 HCC tumor tissues by qRT-PCR analysis, as compared with corresponding normal tissues, *P* < 0.001 (Figures 1B,C).

Next, the experiment of RNase R digestion was carried out to testify the tolerability of hsa_circ_0005785. Total RNA was separated from HCC tissues and treated with RNase R. hsa_circ_0005785 was amplified by our designed primers, and the product was resistant to RNase R digestion. The 18S rRNA, as a linear RNA, was used as a negative control, which could not resist RNase R digestion (Figure 1D). Hsa_circ_0005785's PCR product, including the back-splice junction site, was further checked by Sanger sequencing (Figure 1E), indicating that the primers were specific divergent primers targeting hsa_circ_0005785. Because circRNA lacks a poly A tail, the tissue RNA was reversely transcribed with oligo (dT) primers or random 6-mers and demonstrated with qRT-PCR assays. The 18S rRNA was used as a positive control. The data disclosed that hsa_circ_0005785 could be transcribed by random 6-mer primers effectively rather than by oligo (dT) primers (Figure 1F). These findings revealed that hsa_circ_0005785 owned a complete ring structure without a poly A tail, which was steady in the tumor tissues of HCC patients.

Besides, we also surveyed whether upregulated hsa_circ_0005785 had a relationship with HCC patients' pathological parameters and discovered that it was highly correlated with TNM stage, *P* = 0.018; yet, no obvious correlation was found with other pathological parameters, all *P* > 0.05 (Supplementary Table 1). Additionally, the survival curve was established to assess the prognostic value of hsa_circ_0005785. As expected, hsa_circ_0005785 overexpression rendered HCC patients' poor overall survival time, *P* = 0.007 (Figure 1G). These findings unmasked that upregulated hsa_circ_0005785 played a considerable role in the progression of HCC.

### hsa_circ_0005785 Depletion Repressed HCC Cell Growth and Metastasis *in vitro*

**Figure 2:**
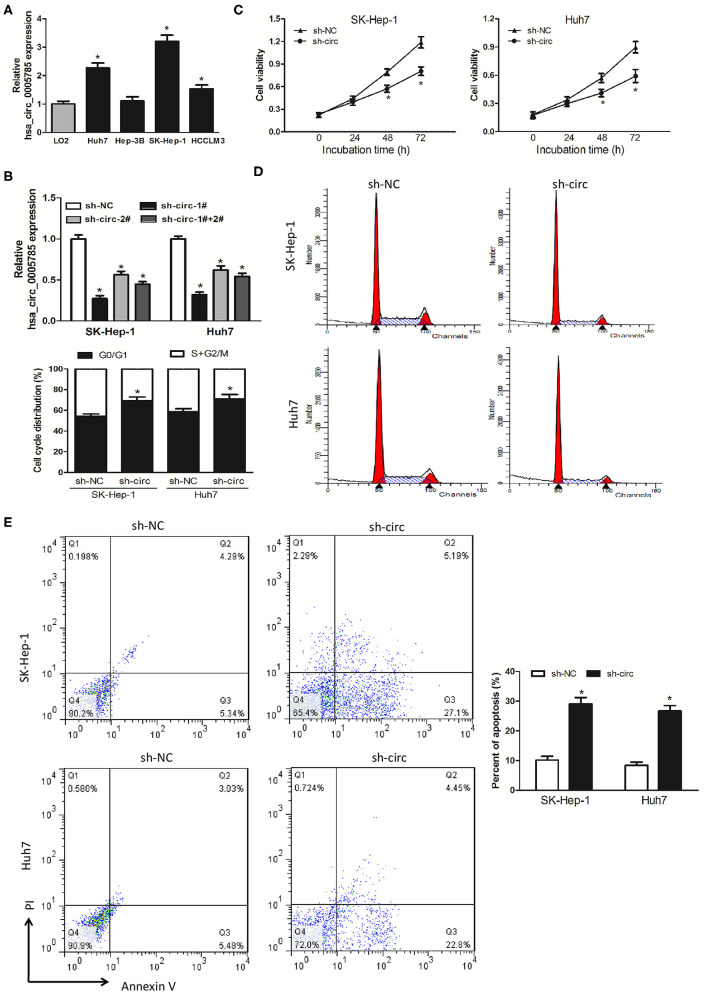
Knockdown of hsa_circ_0005785 inhibits HCC cell growth in intro. **(A)** qRT-PCR analyses of hsa_circ_0005785 expression in 4 HCC cell lines and a normal hepatic cell line LO2. **(B)** qRT-PCR detection of RNA interference efficiency targeting hsa_circ_0005785 was examined in sh-NC (non-targeting control), sh-circ-1#, sh-circ-2#, and sh-circ-#1+#2 transfected SK-Hep-1 or Huh7 cells. **(C)** Cell proliferation was determined in SK-Hep-1 and Huh7 cells after transfection with sh-NC and sh-circ by CCK-8 assay. Detection of cell cycle **(D)** and cell apoptosis **(E)** in SK-Hep-1 and Huh7 cells after transfection with sh-NC or sh-circ by flow cytometry, respectively, *P < 0.05.

We then detected hsa_circ_0005785 expression in 4 HCC cell lines, Huh-7, Hep-3B, SK-Hep-1, and HCCLM3. In comparison with LO2, a normal hepatic cell line by qRT-PCR, hsa_circ_0005785, was dramatically increased in HCC cells, especially in SK-Hep-1 cells and Huh7 cells (Figure 2A). Therefore, both of them were chosen as the representatives for further research. Then, 2 RNA interference vectors targeting hsa_circ_0005785 were constructed, namely, sh-circ#1 and sh-circ#2, and stably transfected into both cell lines, respectively. Both vectors could efficiently suppress hsa_circ_0005785 expression, especially sh-circ#1, showing higher interference efficiency in both cell lines. Besides, sh-circ#1+sh-circ#2 also displayed a similar knockdown efficiency, uncovering that both vectors were hsa_circ_0005785's specific target shRNAs (Figure 2B). Accordingly, we used sh-circ#1 as a representative shRNA for the functional analyses.

CCK-8 assay was performed after sh-circ transfection into HCC cells to estimate cell proliferation. In comparison with the sh-NC group, cell viability was noticeably decreased in both sh-circ-treated SK-Hep-1 cells and Huh7 cells, respectively, *P* < 0.05 (Figure 2C). What is more, cell-cycle distribution was calculated in both sh-circ-transfected cell lines by flow cytometry. As compared with the sh-NC group, both sh-circ treatment groups exhibited that cells in the G0/G1 phase were added, whereas cells in the S+G2/M phase was reduced, *P* < 0.05 (Figure 2D). Besides, cell apoptosis was implemented in both sh-circ-transfected cell lines by flow cytometry. Both sh-circ treatment groups displayed an increase in apoptotic cells, as compared with sh-NC group, *P* < 0.05 (Figure 2E).

**Figure 3:**
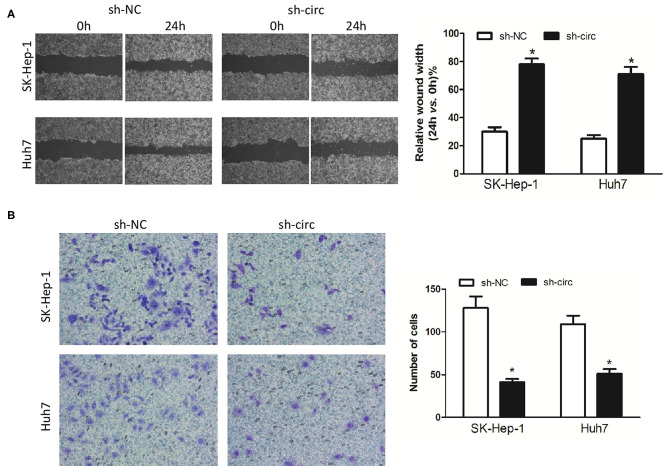
Knockdown of hsa_circ_0005785 inhibits HCC cell metastasis in intro. **(A)** Cell migration activity was performed by wound healing assay in SK-HEP-1 and Huh7 cells after transfection with sh-NC or sh-circ. **(B)** Cell invasiveness was determined by Transwell assay in SK-HEP-1 or Huh7 cells after transfection with sh-NC or sh-circ, *P < 0.05.

Additionally, wound healing assay and transwell assay were applied to evaluate HCC cell migration and invasion, respectively. It turned out as expected that the migrated cells (Figure 3A) and the invasive cells (Figure 3B) in both sh-circ-treated cell lines were obviously dropped, as compared with sh-NC groups, all *P* < 0.05, revealing that hsa_circ_0005785 might own oncogenic properties in boosting the HCC cell metastasis; yet, sh-circ could hold back HCC cell migration and invasion.

### hsa_circ_0005785 Knockdown Suppressed HCC Tumor Growth *in vivo*

**Figure 4:**
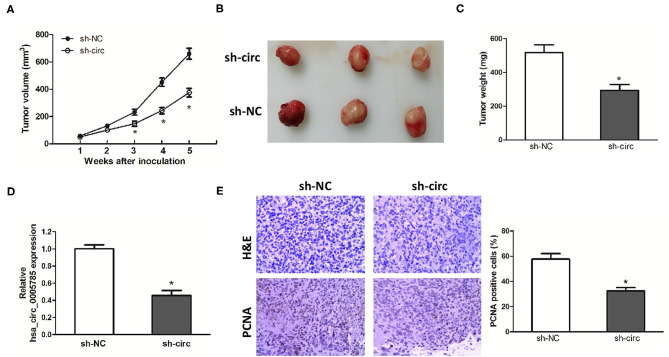
hsa_circ_0005785 knockdown suppresses tumor growth *in vivo*. **(A)** Tumor growth curves measured after injection of SK-HEP-1 cells treated with sh-NC and sh-circ. **(B)** Xenograft tumors were photographed after 5 weeks of inoculation. **(C)** Tumor weight in sh-NC- and sh-circ-treated groups after dissection of the tumor body. **(D)** qRT-PCR detection of hsa_circ_0005785 expression in the resected tumor tissues. **(E)** H&E staining and immunohistochemical staining against the PCNA antibody in sh-NC- and sh-circ-treated groups (× 200), *P < 0.05.

Xenograft models were further established to assess HCC cell growth *in vivo*. The ventral side subcutaneous tissue of athymic mice was injected with SK-Hep-1 cells treated with sh-circ or sh-NC. From 3 to 5 weeks after injection, the tumor size formed in the sh-circ group was smaller and the tumor weight formed in the sh-circ group was lighter than that in the sh-NC group, both *P* < 0.05 (Figures 4A–C). Besides, hsa_circ_0005785 expression by qRT-PCR analysis, HE staining, and the expression of PCNA by immunohistochemical staining were conducted in the resected tumor tissues. Hsa_circ_0005785 expression was apparently reduced in the sh-circ group (Figure 4D); furthermore, PCNA-positive cells in the sh-circ-transfected group were also drastically decreased (Figure 4E), demonstrating that hsa_circ_0005785 depletion could suppress HCC cell growth capacity *in vivo*.

### hsa_circ_0005785 Could Directly Bind to miR-578

**Figure 5:**
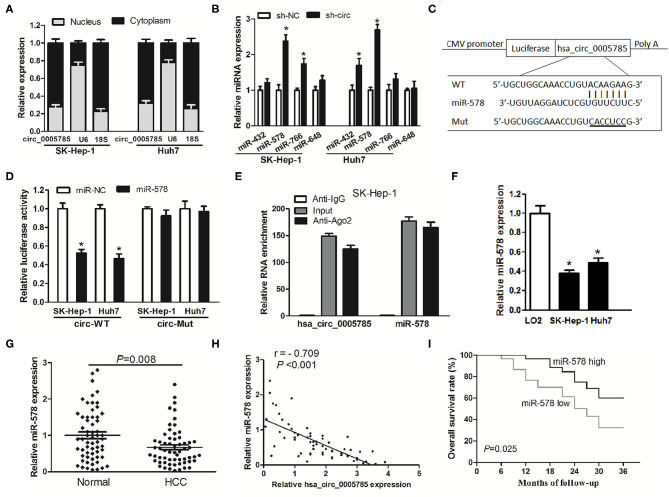
hsa_circ_0005785 directly binds to miR-578. **(A)** qRT-PCR detection of the cytoplasm and nucleus levels of hsa_circ_0005785 in SK-HEP-1 cells and Huh7 cells, 18S rRNA and U6 as cytoplasm and nucleus controls, respectively. **(B)** The expression of 4 predicted targeting miRNAs of hsa_circ_0005785 in sh-NC or sh-circ-treated two HCC cell lines. **(C)** Bioinformatics software predicted the miR-578-binding sequence of hsa_circ_0005785, and its wild-type (WT) or mutant (Mut) miR-578-binding sequence was cloned into pMIR luciferase reporter. **(D)** The luciferase activity was analyzed in two HCC cell lines co-transfected with miR-578 mimic or miR-NC and circ-WT or circ-Mut. **(E)** RIP assay of cellular lysates from SK-HEP-1 cells with Ago2 antibody. hsa_circ_0005785 and miR-578 levels were detected by qRT-PCR. The fold enrichment was presented as Ago2 relative to IgG immunoprecipitates. **(F)** qRT-PCR analysis of miR-578 expression in SK-HEP-1, Huh7, and LO2 cells. **(G)** qRT-PCR detection of miR-578 expression in 60 HCC tissues and paired normal tissues. **(H)** The correlation analysis was conducted between hsa_circ_0005785 and miR-578 levels from 60 HCC tissues. **(I)** Kaplan–Meier curve analysis based on the miR-578 expression level of HCC patients, *P < 0.05.

Accumulating documents have shown that circRNA plays miRNA sponge or ceRNA roles and may control the expression and activity of miRNAs (11, 12, 16). Our qRT-PCR analyses suggested that hsa_circ_0005785 was largely located in the cytoplasm (Figure 5A). Due to the interaction between circRNA and miRNA which usually occurred in the cytoplasm, we conjectured whether hsa_circ_0005785 served as a ceRNA in HCC. Hence, miRNA target sites of hsa_circ_0005785 were predicted by using the online bioinformatic database http://circinteractome.nia.nih.gov/. The expression levels of 4 randomly chosen miRNAs, including miR-432, miR-578, miR-766, and miR-648, were detected by qRT-PCR in sh-circ-treated SK-Hep-1 cells or Huh7 cells. Interestingly, in comparison with the sh-NC group, we found that only miR-578 showed a >2-fold increase in both sh-circ-treated cell lines, whereas the other 3 miRNAs were not or <2-fold changed in sh-circ-transfected groups (Figure 5B), showing that hsa_circ_0005785 might modulate miR-578 expression in HCC cells.

To testify the interaction of miR-578 and hsa_circ_0005785, luciferase reporters inserted with the wild-type sequence of hsa_circ_0005785 (circ-WT) and the corresponding mutant sequence (circ-Mut) were co-treated into SK-Hep-1 cells or Huh7 cells, respectively, together with miR-578 mimic or miR-NC (Figure 5C). Luciferase activity in circ-WT and miR-578 mimic co-transfected two-cell lines was conspicuously decreased. Nevertheless, the luciferase activity was unchanged in circ-Mut and miR-578 mimic co-transfected two-cell lines (Figure 5D). Moreover, RNA binding protein immunoprecipitation (RIP) was conducted in SK-Hep-1 cell extracts with Ago2 antibodies, a key element of RNA-induced silencing complex (RISC). The levels of miR-578 and hsa_circ_0005785 were measured in immunoprecipitates by qRT-PCR analyses. As shown in Figure 5E, when comparing Ago2 pellet groups with IgG control groups, miR-578 and hsa_circ_0005785 were 165- and 124-fold increased, respectively, indicating that hsa_circ_0005785 interacted with miR-578 in Ago2-containing RISC, in accordance with our bioinformatic analysis and the results of luciferase assay.

Afterward, miR-578 expressions in HCC tissues and cells were further surveyed. miR-578 expression was remarkably reduced in SK-Hep-1 cells as well as in Huh7 cells (Figure 5F). Besides, in HCC tissues, miR-578 expression was markedly lower than that in corresponding normal tissues, *P* = 0.008 (Figure 5G), which exhibited an inverse correlation with hsa_circ_0005785 expression, *r* = −0.709, *P* < 0.001 (Figure 5H). Beyond that, our Kaplan–Meier curve disclosed that HCC patients with a lower level of miR-578 had poorer overall survival time, *P* = 0.025 (Figure 5I). Collectively, these findings revealed a ceRNA role of hsa_circ_0005785 in directly binding to miR-578 and regulating its expression in HCC.

### hsa_circ_0005785 Modulated the Expression of APRIL, a mRNA Target of miR-578

**Figure 6:**
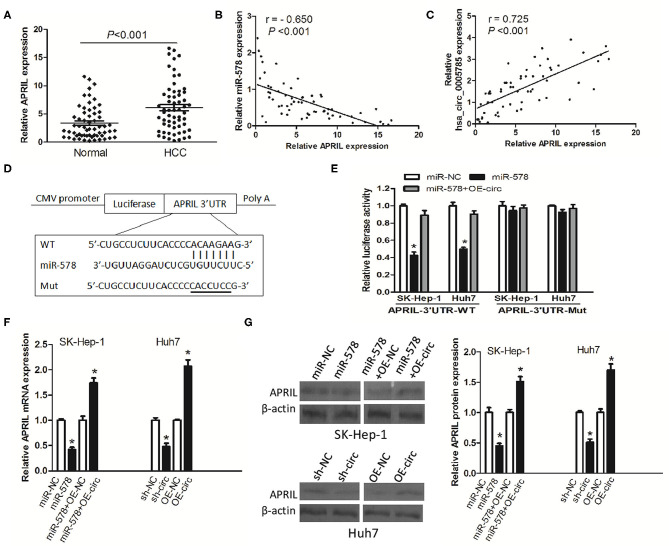
hsa_circ_0005785 regulates the expression of APRIL, an mRNA target of miR-578. **(A)** qRT-PCR detection of APRIL mRNA expression in 60 HCC tissues and paired normal tissues. Correlation analyses of miR-578 vs. APRIL mRNA expression levels **(B)**, and hsa_circ_0005785 vs. APRIL mRNA expression levels **(C)** from 60 HCC tissues. **(D)** Bioinformatics software predicted the miR-578-binding sequence of APRIL mRNA 3′UTR, and its wild-type (WT) or mutant (Mut) miR-578-binding sequence was cloned into the pMIR luciferase reporter. **(E)** Luciferase activity was analyzed in two HCC cell lines co-transfected with miR-NC, miR-578 mimic, or miR-578 mimic+OR-circ, together with APRIL mRNA 3′UTR-WT or 3′UTR-Mut. qRT-PCR **(F)** and Western blot **(G)** analyses of APRIL mRNA and protein expressions in miR-NC-, miR-578 mimic-, miR-578 mimic+OR-NC-, or miR-578 mimic+OR-circ-treated SK-HEP-1 cells, and in sh-NC-, sh-circ-, OE-NC-, or OE-circ-treated Huh7 cells, respectively, *P < 0.05.

To investigate whether hsa_circ_0005785 modulated the expression of miR-578's mRNA target, we found APRIL as miR-578's potential target with online softwares including TargetScan and RNAhybrid. Previous studies have shown that APRIL is always upregulated in many tumor tissues and acts as an oncogene to promote carcinogenesis (17–20). Herein, we also found that in HCC tissues, APRIL mRNA expression was drastically higher than that in corresponding normal tissues (Figure 6A). Furthermore, its expression had an inverse correlation with miR-578 expression and a positive correlation with hsa_circ_0005785 expression in HCC tissues, *r* = −0.650 and 0.725, respectively, both *P* < 0.001 (Figures 6B,C). The results uncovered that APRIL was always co-expressed with hsa_circ_0005785 in HCC; the interaction among hsa_circ_0005785 with miR-578 and APRIL might be biological significance.

To confirm the interaction of miR-578 and the APRIL mRNA 3′-UTR, luciferase reporters inserted APRIL mRNA 3′-UTR wild type sequence (3′-UTR-WT) and the corresponding mutant sequences (3′-UTR-Mut) were co-treated into SK-Hep-1 cells or Huh7 cells, respectively, together with miR-578 mimic, miR-NC, or miR-578 mimic+ overexpressed vector of hsa_circ_0005785 (OE-circ) (Figure 6D). Luciferase activity was substantially decreased after being co-transfected with 3′-UTR-WT+miR-578 mimic into both two cell lines, ensuring that APRIL was a target of miR-578. Notably, when the two cell lines were co-transfected with 3′-UTR-WT+miR-578+OE-circ, the activity of luciferase was partially recovered, due to the existence of hsa_circ_0005785, as compared with the 3′-UTR-WT+miR-578 mimic-treated groups, *P* < 0.05. However, there was no change of luciferase activity in both two cell lines after being co-transfected with 3′-UTR-Mut and any other vector, all *P* > 0.05 (Figure 6E).

Besides, in both miR-578 mimic-treated SK-Hep-1 cells and sh-circ-treated Huh7 cells, the expressions of APRIL mRNA and protein were evidently dropped. Nevertheless, the expressions of APRIL mRNA and protein were apparently raised in miR-578 mimic+OE-circ-treated SK-Hep-1 cells and OE-circ-treated Huh7 cells in comparison with miR-578 mimic+OE-NC and OE-NC groups, respectively, *P* < 0.05 (Figures 6F,G). Taken together, the data strongly indicated a modulating function of hsa_circ_0005785 in derepression of APRIL by binding miR-578, thereby boosting APRIL mRNA and protein expression at the posttranscriptional level.

### hsa_circ_0005785 Reversed the Suppressive Effects of miR-578 on HCC

**Figure 7:**
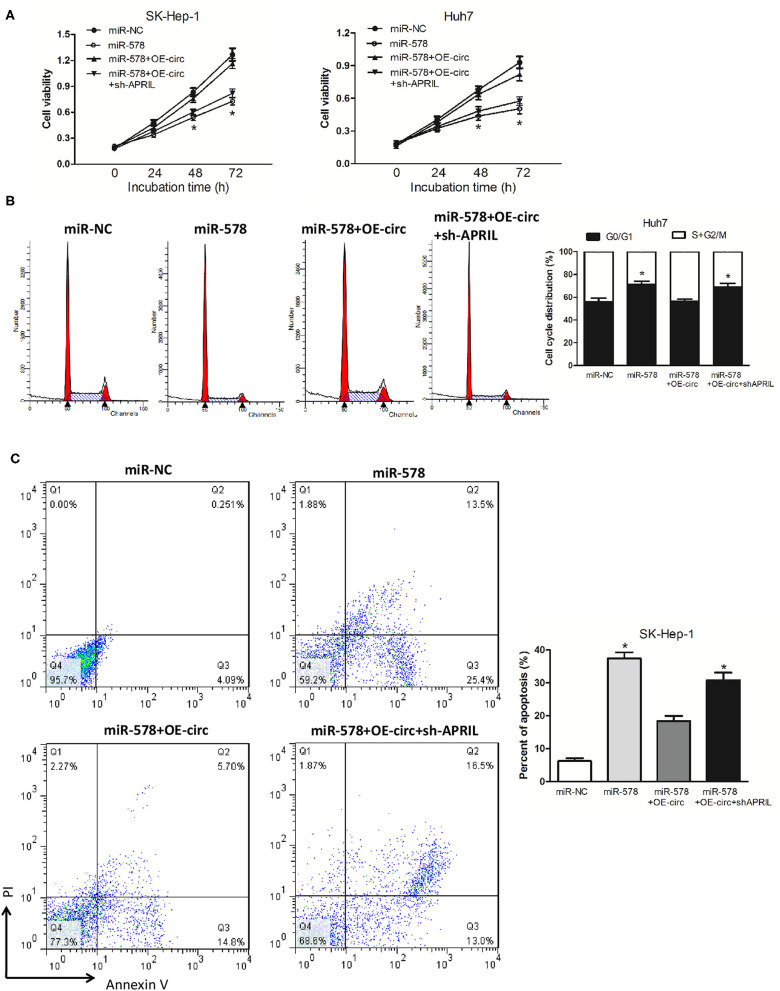
hsa_circ_0005785 reverses the blocking effect of miR-578 on HCC cell growth. **(A)** Cell proliferation was determined in SK-Hep-1 and Huh7 cells after transfection with miR-NC, miR-578 mimic, miR-578 mimic+OE-circ, and miR-578 mimic+OE-circ+ sh-APRIL by CCK-8 assay. Cell-cycle analysis in Huh7 cells **(B)** and cell apoptosis analysis in SK-HEP-1 cells **(C)** after transfection with miR-NC, miR-578 mimic, miR-578 mimic+OE-circ, and miR-578 mimic+OE-circ+sh-APRIL by flow cytometry. *P < 0.05.

We then explored whether hsa_circ_0005785 had the retarding effects on miR-578 activity. In comparison with the miR-NC group, cell viability was observably reduced after miR-578 mimic transfected into both HCC cell lines; yet, in comparison with the miR-578 mimic group, the cell viability in miR-578 mimic+OE-circ co-transfected two-cell lines was substantially enhanced and basically recovered to the original proliferation capacity. As expected, the cell viability in miR-578 mimic+OE-circ+sh-APRIL co-transfected SK-Hep-1 cells or Huh7 cells further fell back dramatically (Figure 7A). The cell-cycle and cell apoptosis assays displayed a G0/G1 phase arrest and an apoptotic cell increase in miR-578 mimic-treated Huh7 cells and SK-Hep-1 cells, respectively. However, we found less G0/G1 phase cells and apoptotic cells in miR-578 mimic+OE-circ co-transfected Huh7 cells and SK-Hep-1 cells, respectively. Inversely, G0/G1 phase progression was further held back and apoptotic cells were further increased in miR-578 mimic+OE-circ+sh-APRIL co-transfected Huh7 cells and SK-Hep-1 cells (Figures 7B,C).

**Figure 8:**
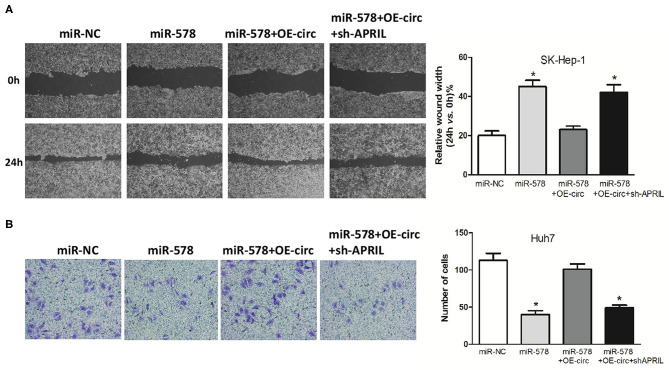
Hsa_circ_0005785 reverses the blocking effect of miR-578 on HCC cell metastasis. Cell migration activity by wound healing assay in SK-HEP-1 cells **(A)** and cell invasiveness by Transwell assay in Huh7 cells **(B)** were performed after transfection with miR-NC, miR-578 mimic, miR-578 mimic+OE-circ, and miR-578 mimic+ OE-circ+sh-APRIL, respectively, *P < 0.05.

Besides, in both miR-578 mimic-treated cell lines, the capabilities of SK-Hep-1 cell migration and Huh7 cell invasion were also depressed. However, the migrated SK-Hep-1 cells and invaded Huh7 cells were evidently added in both miR-578 mimic+OE-circ co-transfected cell lines. Conversely, the SK-Hep-1 cells' migration activity and the Huh7 cells' invasion activity were further dropped in both miR-578 mimic+OE-circ+sh-APRIL co-transfected cell lines (Figures 8A,B). Collectively, these findings unmasked that hsa_circ_0005785 could facilitate malignant progression of HCC via the miR-578/APRIL axis.

## Discussion

Due to the mutation of a large number of susceptible genes, including oncogenes and tumor suppressors, and the effect of assorted external environmental factors, HCC is a complicated malignant tumor whose mechanism is not fully elucidated. What is more, although encouraging advance has been achieved in HCC diagnosis and curative technologies, the overall survival time has not ameliorated as much as we expect in the past decades. Thus, novel biomarkers, which are related to the mechanisms of hepatocarcinogenesis and development, are urgently needed to establish novel strategies to make better clinical practice of HCC (21, 22).

Mounting evidence has disclosed the critical roles of circRNAs in the evolution and development of most human tumors. Also, the expression of circRNAs is associated with specific pathological stages and therapeutic effects, as well as diagnostic and prognostic predictors for all kinds of malignancies, including HCC (23, 24). On the one hand, some circRNAs, such as hsa_circ_0000092 (25), hsa_circ_0056836 (26), and circ_0001955 (27), were upregulated in HCC, which could play oncogenic roles by promoting HCC growth and metastasis. On the other hand, some circRNAs, such as circRNA-5692 (28), hsa_circ_0070269 (29), and circ-ADD3 (30), were downregulated in HCC, which could act as tumor suppressors by inhibiting HCC growth and malignant progression.

In the current research, dysregulated circRNAs were screened with circRNA microarray and was further verified with qRT-PCR. One novel circRNA, namely, hsa_circ_0005785, was frequently upregulated in HCC, but its functions were still unknown. Accordingly, we attempted to explore its potential roles in HCC pathogenesis. Clinically, the upregulated hsa_circ_0005785 was associated with pathological stage, as well as bad prognosis of HCC patients, indicating that it might play a vital role in HCC progression. At the cellular level, specifically, knockdown of the overexpressed hsa_circ_0005785 in HCC cell lines could hold back cell proliferation and metastasis, lead to cell-cycle arrest and an increase in cell apoptosis, and suppress HCC cell *in vivo* growth capacity, revealing that hsa_circ_0005785 had oncogenic properties in accelerating HCC cell malignant phenotype formation.

Then, the molecular mechanisms of hsa_circ_0005785 promoting malignant progression of HCC were further investigated. Our subcellular analysis displayed that the location of hsa_circ_0005785 was always in the cytoplasm. Hence, we wondered whether hsa_circ_0005785 served as ceRNA or miRNA sponge. Thereupon, our bioinformatic prediction and qRT-PCR analysis confirmed the function of hsa_circ_0005785 in modulating miR-578 expression in HCC cells. Moreover, luciferase activity experiment and RIP assay further verified that hsa_circ_0005785 served as a miRNA sponge, which existed in RISC via interaction with miR-578. Ji et al. found that circ_001621 could quicken osteosarcoma cell growth and metastasis through the miR-578/VEGF axis, providing new therapeutic targets for osteosarcoma (31). Zhou et al. reported that circular RNA-ZBTB44 participated in choroidal neovascularization progress via the miR-578-VEGFA/VCAM1 axis, supplying new clinical practice in neovascularization-related diseases (32). Besides, miR-578 was also downregulated in breast cancer and related to tumor cell adhesion and angiogenesis (33). Nevertheless, the exact function and pathogenesis of miR-578 in HCC are still elusive. We discovered that miR-578 expression was decreased and demonstrated an inverse correlation with hsa_circ_0005785 expression in HCC tissues, showing a potential tumor suppressor in HCC.

After that, we further investigated whether hsa_circ_0005785 might modulate certain mRNA expression, as miR-578's target. Our bioinformatic prediction and luciferase activity experiment testified that APRIL served as miR-578's potential target. Garcia-Castro et al. found that APRIL could advance the breast cancer promotion and was related to invasive breast cancer (34). Dou et al. reported that APRIL was upregulated in non-small cell lung cancer and could promote cancer malignant phenotype formation (35). Our previous studies had disclosed that APRIL was overexpressed in colorectal cancer, and its depletion induced colorectal cancer cell apoptosis and cell-cycle arrest (36, 37). Additionally, we also discovered that recombinant human APRIL protein could accelerate HCC cell proliferation (38). Herein, overexpressed APRIL was further found to have an inverse correlation with miR-578 expression in HCC tissues, whereas it had a positive correlation with hsa_circ_0005785 expression. Besides, hsa_circ_0005785 regulated the derepression of APRIL by binding miR-578, thereby boosting APRIL mRNA and protein expression at the posttranscriptional level. These findings disclosed that the interaction among hsa_circ_0005785, miR-578, and APRIL might be a biological significance in HCC.

Additionally, we further explored whether hsa_circ_0005785 had the suppressive influence on miR-578's activities. As expected, miR-578 restrained HCC cell proliferation and metastasis, whereas hsa_circ_0005785 overturned the suppressive influence of miR-578 on HCC through derepression of APRIL expression, revealing that hsa_circ_0005785 could facilitate malignant progression of HCC via the miR-578/APRIL axis.

Taken together, our present data highlights that overexpressed hsa_circ_0005785 may serve as a hopeful biomarker for HCC clinical practice. Furthermore, it also plays as an oncogene to boost the HCC malignant process via the miR-578/APRIL axis. Importantly, the hsa_circ_0005785/miR-578/APRIL regulatory pathway contributes to the attractive diagnostic and therapeutic strategy for HCC.

## Data Availability Statement

The original contributions presented in the study are publicly available. This data can be found here: https://figshare.com/s/6d9d372ccb7d638f6d65.

## Ethics Statement

The animal study was reviewed and approved by the Animal Ethics Committee of Nantong University. The studies involving human participants were reviewed and approved by the Ethics Committee of Affiliated Hospital of Nantong University. The patients/participants provided their written informed consent to participate in this study.

## Author Contributions

SJ, LC, and FW designed the study. MK, BZ, RL, and FB performed experiments and collated the data and carried out data analyses. AW and YL contributed to drafting the manuscript. All authors have read and approved the final submitted manuscript.

## Conflict of Interest

The authors declare that the research was conducted in the absence of any commercial or financial relationships that could be construed as a potential conflict of interest.
