# Supplementary material for: Upregulated hsa_circ_0005785 Facilitates Cell Growth and Metastasis of Hepatocellular Carcinoma Through the miR-578/APRIL Axis
Source: Front Oncol. 2020 Aug 19;10:1388. doi: 10.3389/fonc.2020.01388 (PMC7466587; doi:10.3389/fonc.2020.01388)
Supplement: Supplementary file 1 [file Data_Sheet_1.docx]

**Supplementary Table 1:** Relationship of hsa_circ_0005785 expression with clinicopathological features in HCC patients

|  | Cases | hsa_circ_0005785 expression | | |  |
| --- | --- | --- | --- | --- | --- |
| Variables | (n=60) | High (n=30) | Low (n=30) | *P* value | |
| Age (year) |  |  |  | 0.439 | |
| ≤58 | 30 | 13 | 17 |  | |
| >58 | 30 | 17 | 13 |  | |
| Gender |  |  |  | 0.353 | |
| Male | 55 | 29 | 26 |  | |
| Female | 5 | 1 | 4 |  | |
| HBV infection |  |  |  | 0.267 | |
| Positive | 41 | 23 | 18 |  | |
| Negative | 19 | 7 | 12 |  | |
| Hepatocirrhosis  Present  Absent  Serum AFP (ng/mL)  ≤200  >200  Tumor size (cm) | 35  25  13  47 | 19  11  4  26 | 16  14  9  21 | 0.601  0.209  0.187 | |
| ≤5 | 24 | 9 | 15 |  | |
| >5 | 36 | 21 | 15 |  | |
| Tumor differentiation |  |  |  | 0.110 | |
| Well + Moderate | 37 | 15 | 22 |  | |
| Poor | 23 | 15 | 8 |  | |
| TNM stage |  |  |  | 0.018* | |
| I-II | 26 | 8 | 18 |  | |
| III-IV | 34 | 22 | 12 |  | |

Fisher’s exact test, **P*<0.05

**Supplementary Table 2:** Primer sequence for qR-PCR

| Target Sequence | | |
| --- | --- | --- |
| hsa_circ_0005785 | forward | 5’-GTCGCTTAGATTGTTATGAAGTGAG-3’ |
|  | reverse | 5’-AGAGAGAATCTGCATGATACACCAA-3’ |
| APRIL | forward | 5’-ACTCTCAGTTGCCCTCTGGTTG-3’ |
|  | reverse | 5’-GGAACTCTGCTCCGGGAGACTC-3’ |
| miR-578 | forward | 5'-GTGCAGGGTGTTAGGA-3′ |
|  | reverse | 5'-GAAGAACACGTCTGGT-3′ |
| miR-432 | forward | 5'-AACGAGACGACGACAGAC-3′ |
|  | reverse | 5'-CTTGGAGTAGGTCATTGGGT-3′ |
| miR-766 | forward | 5'-AACAAGAGGAGGAATTGGTGCTG-3' |
|  | reverse | 5'-CAGTGCAGGGTCCGAGGT-3' |
| miR-648 | forward | 5'-CACAGACACCTCCAAGTG-3' |
|  | reverse | 5'-CCCTCACTTCCGACTAAG-3' |
| 18S rRNA | forward | 5'-GTAACCCGTTGAACCCCATT-3′ |
|  | reverse | 5'-CCATCCAATCGGTAGTAGCG-3′ |
| U6 | forward | 5'-AGAGCCTGTGGTGTCCG-3′ |
|  | reverse | 5'-CATCTTCAAAGCACTTCCCT-3′ |

**Supplementary Table 3:** shRNA sequences of target genes

| Target genes | Sequences |
| --- | --- |
| sh-hsa_circ_0005785 (sh-circ#1) | 5′- TGAAGTGAGTTATTCTCCCCA-3′ |
| sh-hsa_circ_0005785 (sh-circ#2) | 5′-GTTATGAAGTGAGTTATTCTC-3′ |
| sh-APRIL  sh-NC | 5’-CAACCTTCTTCCCTTCTGC-3’  5’-TTCTCCGAACGTGTCACGT-3’ |
